# PCIF1, the only methyltransferase of N6,2-O-dimethyladenosine

**DOI:** 10.1186/s12935-023-03066-7

**Published:** 2023-10-01

**Authors:** Yuting Wu, Xi Pu, Sihui Wu, Yiran Zhang, Shengqiao Fu, Haowen Tang, Xu Wang, Min Xu

**Affiliations:** 1 Department of Gastroenterology, Affiliated Hospital of Jiangsu University, Jiangsu University, Zhenjiang, 212001 Jiangsu China; 2 Digestive Disease Research Institute of Jiangsu University, Zhenjiang, 212001 Jiangsu China; 3 Department of Radiation Oncology, Institute of Oncology, Affiliated Hospital of Jiangsu University, Zhenjiang, 212001 Jiangsu China

**Keywords:** PCIF1, N6,2-O-dimethyladenosine, Immunity, PD-1

## Abstract

N6-methyladenosine(m6A), is the most abundant post-transcriptional modification of mRNA in biology. When the first nucleotide after the m7G cap is adenosine, it is methylated at the N6 position to form N6,2-O-dimethyladenosine (m6Am). m6Am is a reversible modification located at the first transcribed nucleotide, which is present in about 30% of cellular mRNAs, thus m6Am can have a significant impact on gene expression in the transcriptome. Phosphorylated CTD interaction factor 1(PCIF1), the unique and specific methyltransferase of m6Am, has been shown to affect mRNA stability, transcription, and translation. Several studies have shown that PCIF1 is clearly associated with tumor, viral, and endocrine diseases. Moreover, PCIF1 may be related to the tumor microenvironment, immune cell typing, and programmed cell death protein 1(PD-1) drug resistance. Here, we summarize the mechanism of PCIF1 involvement in mRNA modifications, and outline m6Am modifications and diseases in which PCIF1 is involved. We also summarized the role of PCIF1 in immune and immune checkpoint blockade(ICB) treatment, and predicted the possibility of PCIF1 as a biomarker and therapeutic target.

## Introduction

m6A is the most abundant post-transcriptional modification in nature, followed by m6Am modification. These two reversible modifications are the most common [1]. Almost every post-transcriptional process, including mRNA splicing, translocation, transcription, stability, and translation, involves the m6A alteration. m6A modification is also involved in cell division, differentiation and play an important role in cancer proliferation, neurological function[2]. A triphosphate bond holds the 7-methyl-guanosine (m7G) cap, which generally appears at the 5' end of eukaryotic mRNA, to the rest of the mRNA. When 2-O-methyl-adenosine is the first nucleotide after the m7G cap, it will undergo further methylation at its N6 position to create the m6Am modification [3, 4]. 30% of cellular mRNAs contain m6Am at the first transcribed nucleotide, thus m6Am can have a significant impact on gene expression in the transcriptome [5].

PCIF1, also known as CAPAM, has been shown to be the only mammalian mRNA m6Am methyltransferase in several studies [6]. PCIF1 was first discovered for its capacity to bind specifically to RNA polymerase II's phosphorylated C-terminal domain (CTD) via its WW domain [6, 7]. Human PCIF1 contains 704 amino acids and the N-terminal WW domain is involved in protein–protein interactions [8]. PCIF1, which is mostly found in the nucleus, is responsible for producing the m6Am modification in new transcripts. m6Am was detected after treatment with the uncapped enzyme, suggesting that m6Am was limited to the first nucleotide adjacent to the m7G cap of the mRNA [3, 8, 9]. Here, we summarize the mechanisms by which PCIF1 regulates transcripts and in various diseases. We also summarized the role of PCIF1 in immune and ICB treatment, and predicted the possibility of PCIF1 as a biomarker and therapeutic target.

## The structure of PCIF1

PCIF1, also known as CAPAM, is the only “writer” of m6Am. PCIF1 is also the only cap specific adenosine N6 methyltransferase located in the nucleus [8]. Transcripts can be detected in 16 different tissues using labeled PCIF1 cDNA, indicating that PCIF1 is widely expressed in mRNA and is highly conserved during evolution [6, 7, 10].

The spatial structure of PCIF1 from the N-terminal to the C-terminal is a WW domain rich in tryptophan, an extension consisting of 98 amino acid residues, a helical domain (HD), and an MTD, forming the “WW-linker-HD-MTD” structure [11]. Human PCIF1 has a molecular weight of 81 kDa and contains a long open reading frame, encoding a polypeptide containing 704 amino acids. At the N-terminal, there is a WW domain. It is a short conserved region of approximately 40 amino acids, folded into a stable triple chain antiparallel. There are two tryptophan residues in this domain, spaced 20–23 amino acids apart [8]. Through improved Western blot analysis, we found that the WW domain can directly bind to the Ser5 phosphorylated C-terminal domain (pCTD) of RNA polymerase II. Radioactivity measurements showed that the binding of WW domains to pCTD was 20 times greater than that to CTD, demonstrating that phosphorylation of CTD resulted in a considerable increase in the binding affinity of PCIF1 WW domains to CTD. The WW domain of PCIF1 is highly homologous to the WW domain of human peptidylprolyl isomerase (PPIase) Pin1 (Pin1-WW). They have similar structures and functions [6]. The PCIF1 WW domain specifically interacts with Ser5 phosphorylated CTD, while the Pin1 WW domain interacts with both Ser5 and Ser2 peptides. Pin1 has the ability to control Pol II activity in the transcription cycle and cell cycle as well as the degree of phosphorylation of the CTD. SCP1 is the phosphatase of CTD, which inhibits the dephosphorylation of CTD in a dose dependent manner. Both PCIF1 and Pin1 effectively inhibited the CTD phosphatase activity of SCP1 in vitro, revealing that specific and direct binding by the PCIF1 WW domain can influence the phosphorylation status of Pol II. Using a luciferase assay to examine the trans-activation activity of cell transcription activating factors, it was found that PCIF1 inhibits trans-activation, suggesting that PCIF1 negatively regulates Pol II [7, 10, 12]. The main function of the PCIF1 WW domain is to participate in protein–protein interactions without affecting the activity of PCIF1 methyltransferase in vitro, and it does not play a role in N6 methylation reactions [6, 13].

The HD of PCIF1 is a helical domain located near the MTD catalytic ring. HD covers two triple helical bundles(α1-α6-α8 and α4-α5-α6), a four helical beam(α1-α2-α3-α6) and two β- folds(β1-β2 and β3-β 4-β5) [14]. This domain has no homology with other domains. 15% of the residues in HD are positively charged. The majority of them are surface-exposed and substantially preserved in animals [8]. Mutations in these residues can reduce the activity of MTase. The function of helical domains may be related to protein stability [6].

**Fig. 1:**
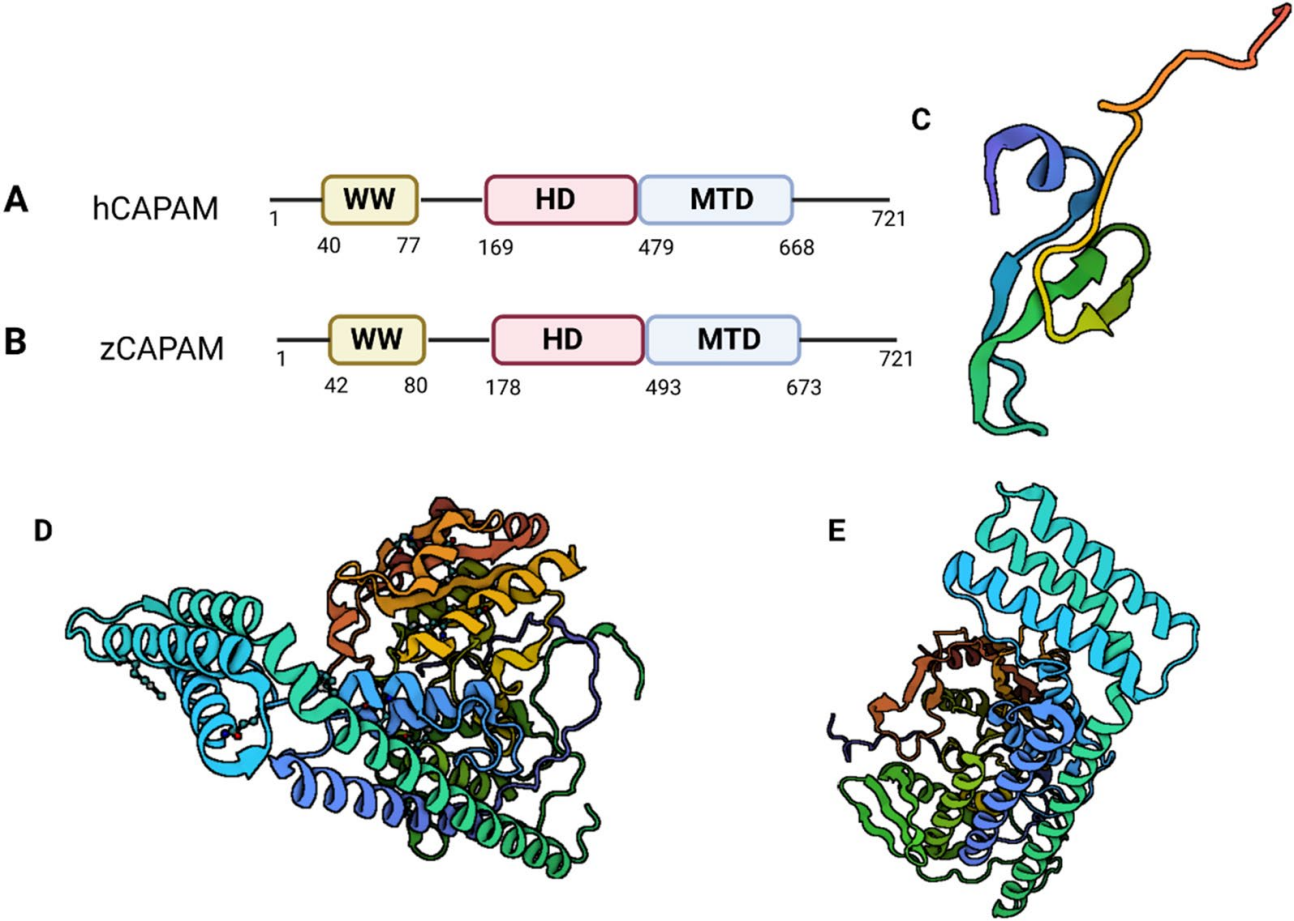
The structure of PCIF1:**A**: Domain organization of human CAPAM(hCAPAM). **B**: Domain organization of zebrafish CAPAM(zCAPAM). **C**: The WW domain of hPCIF1(PDB 2JX8). **D**: Structure of human CAPAM(PDB 6IRX).**E**: Structure of zebrafish CAPAM(PDB 6IRV). (The 3D protein structure map was derived from PROTEIN DATA BANK)

The MTD of PCIF1 presents a classic Rossmann fold. This structure can also be found in all proteins that are functional m6A RNA MTases [6, 7]. The structure includes a substrate binding ring (SBL: P612-N638), a catalytic ring (CL: N553-C557), and an active site ring (ASL: F479-P494). The substrate binding ring depends on the degree of variation of the substrate [10]. The inactivation of PCIF1 residue mutations in vitro and in vivo proves that the catalytic ring catalyzes the conservative NPPF motif. Mutants of PCIF1 are often generated by mutations in asparagine 553 and phenylalanine 556 to alanine (NPPF/APPA) or serine and glycine (NPPF/SPPG) [15]. This is crucial in verifying PCIF1-mediated m6Am activity. (Fig. 1).

## PCIF1 is considered a cap-specific N6 methyltransferase of m6Am

An m7G cap is present at the 5' end of mRNA. It can enlist cap-binding proteins that are involved in mRNA transcription or translation, shield mRNA from immunological reactions, and guard against destruction [16]. The structure of mRNA caps varies among species. In mammals, the cap is expressed as m7G (50) ppp (50) Xm, which can form a triphosphate connection with the other mRNA to connect them [8].

m6Am modification is a reversible modification that is second only to m6A and exists in 30% of mRNAs. It can be detected in zebrafish, human cells, and mice, but it is not present in samples of Schizosaccharomyces, nematodes, or fruit flies [5, 11]. m6Am modification is a very rich and evolutionarily conserved modification in vertebrate animals, and its function is still unclear [3]. Some studies have shown that m6Am modified genes are mainly enriched in various obesity and metabolism-related processes [18]. Researchers have identified key metabolic genes for m6Am methylation in obese mice, including two m6Am specific methylated fatty acid binding proteins FABP2 and FABP5 [18]. FTO is significantly upregulated in obese mice. FTO participates in the demethylation of m6Am and affects the protein level of its m6Am methylation target [19].Normal cells maintain the same growth as m6Am-deficient cells. However, under oxidative stress, cells lacking m6Am exhibit a slower growth rate, indicating that m6Am is involved in the response of cells to external oxidative stress [3, 20]. Genes with elevated m6Am levels are associated with endoplasmic reticulum (ER) stress responses. Specifically, the m6Am levels of CHOP and ATF3 mRNA are significantly increased [21]. In addition, the metabolism of cells is also significantly influenced by hypoxia. Hypoxia can increase the mRNA and protein levels of the hypoxia-inducible factor (HIF)-1 target gene GLUT1, thus increasing glucose transport. Increased levels of m6Am in GLUT1 mRNA suggest that m6Am modification may play a role in hypoxic adaptation and glucose metabolism [21, 22]. In mice, the disappearance of m6Am levels inhibits highly expressed transcripts in meiotic spermatocytes and haploid round sperm cells. The decrease in these transcripts should have affected sperm production, but it was observed that the fertility of the mice was not affected, while weight loss occurred [3, 11]. It has been shown that the Drosophila homolog of PCIF1 catalyzes death and is associated with fertility. The researchers observed a significant increase in gene expression of the mitochondrial ATP synthase complex in PCIF1 mutant female flies [23].

m6Am is crucial to the fate of mRNA. There are similar chemical reactions between m6Am and m6A modifications, but there are also some differences. (1) Adenosine is methylated at the 2'-OH position to form 2'-O-methyladenosin, which is then further methylated to m6Am.Adenosine is the first nucleotide next to the m7G cap structure [10]. (2) Different writers: PCIF1 is a m6Am-specific methyltransferase, while METTL3,METTL14,WTAP, and KIAA1429 methyltransferase complexes participate in m6A modification [2, 10]. (3) Different demethylases: m6A can be demethylated by ALKBH5 and FTO, but ALKBH5 is considered first, while m6Am is only demethylated by FTO. FTO preferentially demethylates m6Am [9, 19]. (4) Different locations: m6A modifications present on various mRNAs are typically concentrated around the termination codon, while m6Am often occurs near the mRNA cap [2, 8, 17, 24] (Table 1).

**Table 1:** The difference between m6A and m6Am modification

	m6A	m6Am
Definition	N6-methyladenosine	N6,2-O-dimethyladenosine
Chemical structure		
Position	Termination codon	Near the cap
Methyltransferase	METTL3, METTL14, WTAP and KIAA1429 methyltransferase complex	PCIF1
Demethylase	ALKBH5 、FTO	FTO
Physiological function	Embryonic development, neurogenesis, circadian rhythm, stress response and tumorigenesis	Unknown

PCIF1 is located in the nucleus and colocalized with endogenous RNAP IIO in the nucleus. In HeLa cells, PCIF1 is more abundant in the chromatin portion than in the nucleus. Immunofluorescence revealed that PCIF1 was colocalized with H3K4me3, which is a marker of transcriptionally active gene promoters.PCIF1 exists in the chromatin region of the nucleus, and this region has transcriptional activity [6]. PCIF1 has been shown to be the cap specific adenosine N6-methyltransferase of m6Am. The WW domain of PCIF1 interacts with the Ser5 phosphorylated CTD of RNAPII to form m6Am during early transcription. The m7G cap is necessary for PCIF1 N6 methylation, and the Am residue on the decapped mRNA cannot be successfully converted to m6Am [26]. PCIF1 specifically recognizes and binds directly to the m7G cap through its helical domain. It prioritizes N6-methylated 2'-O-methyladenosine rather than the internal adenosine of mRNA, and this methylation requires the catalytic domain of PCIF1. Mutant PCIF1 without catalytic activity cannot generate m6Am. In addition, PCIF1 had no effect on the total m6A level. After PCIF1 KO, the m6A level did not change, and m6Am completely disappeared, indicating that m6A modification does not require PCIF1, and the m6Am level had no effect on m6A [6, 16].

## Mechanism of PCIF1 in regulating mRNA

PCIF1 catalyzes not only RNA, but also double stranded DNA, single stranded DNA, and DNA/RNA hybrid chains. Its catalytic ability ranges from high to low: ssRNA, RNA/DNA hybrids, ssDNA, and dsDNA [27]. Here, we summarize the role of PCIF1 modification in mRNA transcription, stability, and translation [28] (Table 2).

**Table 2:** Mechanism of PCIF1 in regulating mRNA: This table summarizes all studies on the role of PCIF1 in mRNA modification, listing the authors, cell lines, mechanisms of mRNA modification, and methods for identifying m6Am

Researches	Cell line	Function of PCIF1 on mRNA	Method	References
Radha Raman Pandey	Mouse Tissues	Increase mRNA stability	m6Am-Exo-Seq	[3]
Konstantinos Boulias	HEK293T	Increase mRNA stability	miCLIP	[5]
Lingling Wang	HCT116 cells HT29 cells	Increase mRNA stability	m6Am-exo-seq	[29]
Jan Mauer	HEK293T cells	Increase mRNA stability	miCLIP	[30]
Qiong Zhang	HeLa cells 293FT cells	Increase mRNA stability	m6Am-Exo-Seq	[31]
Sendinc	MEL624 cells	Suppress cap dependent translation	m6Am-Exo-Seq LC–MS/MS	[9]
Wei Zhuo	AGS cells	Suppress mRNA translation	m6Am-seq	[32]
Shinichiro Akichika	HEK293T cells	Promote cap dependent translation	miCLIP	[8]

### PCIF1 in gene transcription/stability

Raman Pandey found that cap specific m6Am modification can promote the stability of mouse tissue transcripts [3]. In PCIF1 mutant mice, Y-chromosome related genes tend to decline. 90% of these downregulated genes use adenosin as the TSS nucleotide, indicating that the majority of downregulated genes are associated with m6Am. Using ribosomal analysis, it was shown that the translation level of most transcripts in the tissues after PCIF1 mutation did not change. However, in mutated brain tissue, some pseudogenes and uncertain predictive genes have significant changes, but these changes are not related to TSS [3]. Ai Sugita observed that in HeLa cells, PCIF1 is located in the chromatin of the nucleus and recruits the promoter regions of certain genes in a transcription dependent manner [17]. It functions as a non- methyltransferase. These genes include constitutively expressed GAPDH, MYC, and inducible FOS genes. Although PCIF1 binds to the promoters of these genes, there was no significant change in the mRNA levels of GAPDH and MYC after knockdown of PCIF1 with siRNA. This indicates that PCIF1 has a limited impact on mRNAs transcriptome [17]. Konstantinos Boulias found that m6Am preferentially appears in highly stable and abundant transcripts [5]. These transcripts are divided into lower and upper halves of gene expression. The lower half of the genes show a decreased half-time period, while the upper half of the genes are not affected. Ribosome analysis showed that there was no change in m6Am-related proteins after PCIF1 KO. This indicates that depletion of PCIF1 does not significantly affect mRNA translation in HEK293T cells [5]. Wang found that FOS is a downstream target gene of m6Am modification in colorectal cancer [29]. Knocking out PCIF1 in HCT116 and HT29 cells results in a decreased half-life period of FOS mRNA. This shows that PCIF1 mediates the stability of FOS mRNA [29]. Jan Mauer found that mRNA starting with m6Am is more stable in HEK293T cells [30]. Its half-life increases approximately by 2.5 h, exhibiting higher transcriptional levels and higher translation efficiency. The reason may be that m6Am has an antagonistic effect on the capping enzyme DCP2. Therefore, mRNA starting with m6Am is not affected by DCP2-dependent degradation, thus increasing the stability of mRNA. MicroRNA(miRNA) also mediate mRNA degradation, which is an endogenous degradation pathway. m6Am reduces the degradation caused by miRNAs, which can also enhance the stability of mRNA [30, 33, 34]. Zhang found that in HeLa and 293FT cells infected with HIV, PCIF1 increases the stability of EST1 mRNA and limits the replication of HIV. In PCIF1 KO cells, the expression of ETS1 mRNA decreased significantly, and the half-life decreased from 3.2 h to 2.4 h, indicating that PCIF1 mediated the stability of ETS1 mRNA. In T cells, PCIF1 is overexpressed, and ETS1 expression increases more than twofold [31].

### PCIF1 in gene translation

Sendinc observed that mRNA stability was not affected in PCIF1 knockout MEL624 cells [9]. However, m6Am specifically inhibits cap dependent translation. Quantitative proteomic analysis was performed on control cells and PCIF1 KO cells. It was found that a group of transcriptional protein levels significantly decreased while the transcriptional level remained unchanged [9]. Wei also found that in gastric cancer cells, the half-life of AGS cells with PCIF1 KO did not change, indicating that PCIF1 did not affect mRNA transcription and stability changes [32]. However, cells beginning with m6Am showed a decrease in GFP signaling, while polyribosomal analysis showed no significant change [32]. In AGS cells, PCIF1 inhibits mRNA translation, but does not affect the distribution of polyribosomes. This reveals the translation inhibitory effect of PCIF1 [32]. However, Akichika reached the opposite conclusion [8]. After knocking out PCIF1, the translation efficiency of HEK293T cells was downregulated, and the downregulated genes showed m6Am enrichment. These genes are associated with mRNA translation, transport, and metabolism, demonstrating that PCIF1 promotes the translation level of capped mRNA. Ribosomal analysis showed that PCIF1 KO did not affect the distribution of ribosomes. The translation initiation factor eIF4E directly recognizes the cap structure of mRNA, thereby promoting cap dependent translation. The authors examined the binding ability of eIF4E to m6Am mRNA using electrophoretic mobility shift analysis, and found that m6Am had no effect on the binding of eIF4E to the cap structure. This finding indicates that PCIF1 promotes cap- dependent translation through non-eIF4E methods [8].

In summary, the effect of PCIF1 on mRNA is uncertain and researchers are aware of this problem. The reason may be related to the following. First, Researches use different methods for identifying m6Am. Due to the lack of specific m6Am antibodies, distinguishing m6A from m6Am has always been a difficult issue. The peak caused by m6Am usually has a unique shape, which is manifested by a significant decrease in reading at the annotated A starting TSS [9, 21]. However, m6A that appears near the TSS can also exhibit similarly shaped peaks. Therefore, sometimes m6A is mistaken for m6Am, resulting in false positives [21]. A variety of methods have emerged for identifying m6Am levels, including m6Am-Exo-Seq, miCLIP, m6Am-seq, CAPturAM, and others. Different methods may result in differences in m6Am levels. Next, different cell lines as well as different tissue types may also exhibit differences. Studies have shown that the identity and methylation status of the first nucleotide are highly dependent on the cell line, so different cells may show differences in m6Am levels [28, 35].Moreover, the transcription as well as translation of mRNA are affected by a variety of factors. It has been shown that the presence of m6Am reduces the activity of DCP2, thereby enhancing mRNA stability. But DCP2 is not the only decapping enzyme, and m6Am levels may affect other decapping behaviors. In addition, decapping activity is also influenced by RNA structure, RNA-binding proteins, and the cellular surroundings in vivo [30]. The translation initiation factor eIF4E activates translation initiation by recognizing mRNA cap structure. PCIF1 promotes cap-dependent translation through a non-eIF4E approach and PCIF1 has little effect on eIF4E-cap interactions [8]. Hence, there may be other factors acting on eIF4E that affect mRNA stability. In addition, the 2'-O-methylation modification in the second nucleotide of the cap structure also affects translation efficiency. The introduction of a single methyl group at the second transcribed nucleotide in a transcript that begins with an adenine results in reduced translation efficiency [28].

## PCIF1 plays an important role in different diseases

**Fig. 2:**
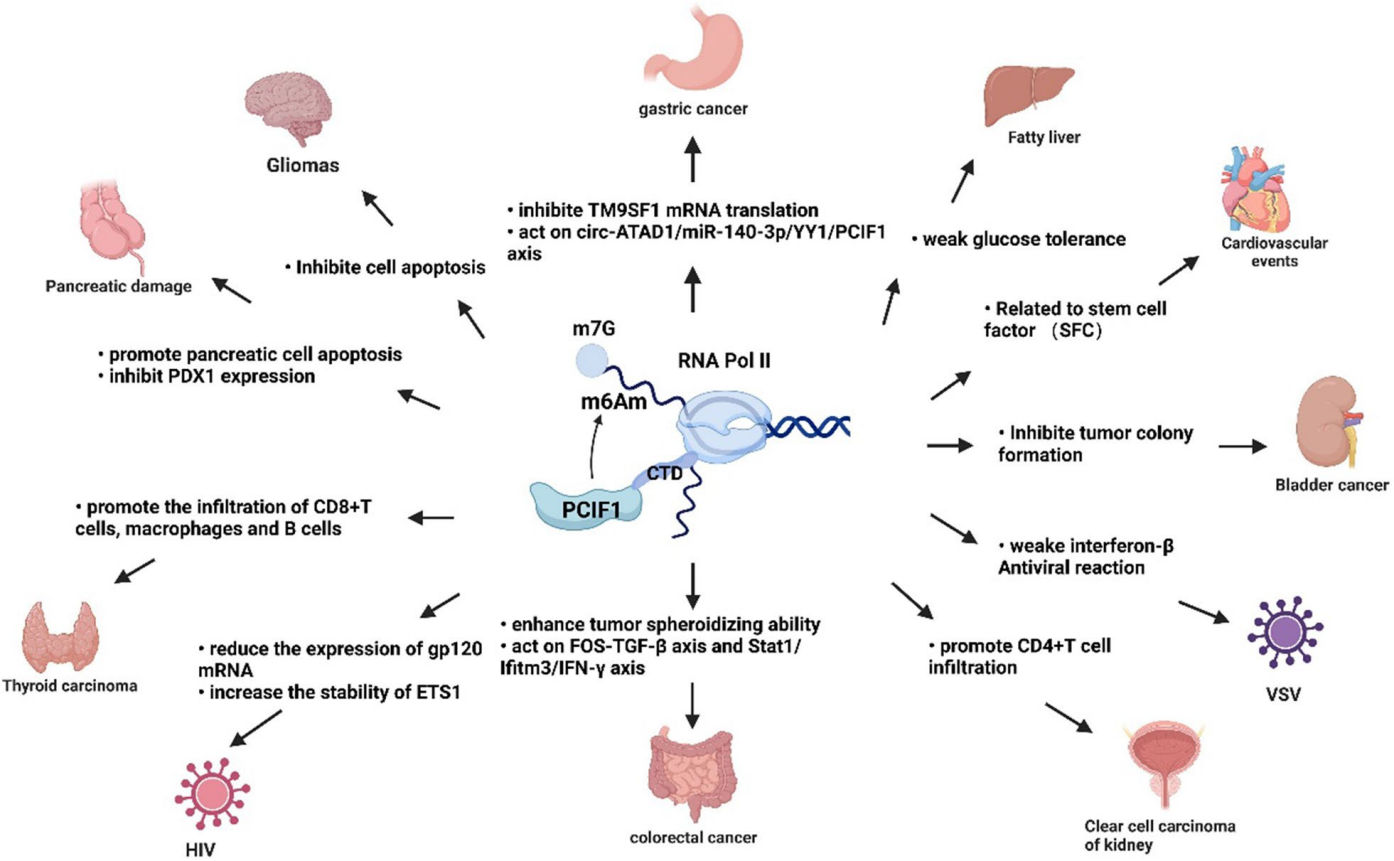
PCIF1 plays an important role in different diseases and its specific mechanisms

PCIF1 has been proven to play an important role in a variety of diseases, including cancers, viral infection, growth, development and endocrine diseases. Next, we summarize the changes in PCIF1 indicators in different systems and their mechanisms. (Fig. 2) In addition, we also summarize PCIF1 mediated m6Am targets. For targets that were verified, we summarized the cell lines and biological functions (Table 3).

**Table 3:** verified m6Am targets of PCIF1

Targets	Cell lines	Diseases	Functions	References
TM9SF1	AGS cells	Gastric cancer	Promote cell invasion, proliferation and tumor metastasis	[32]
ACE2 TMPRSS2	Calu-3/NHBE/Huh7 cells	SARS-CoV-2	Facilitate SARS-CoV-2 infection	[36]
ETS1	HeLa/293FT cells	HIV	Restrict HIV replication	[31]
VSV	HeLa/A549/Vero CCL81/ BsrT7/5 cells	VSV	Enhances viral replication	[37]
PDX1	HEK293T/Min6 β cells	Glucose homeostasis	Improve glucose tolerance and normalizes β cell mass	[38]
FOS	HCT116/HT29 cell	Colorectal cancer	Promote cell proliferation, migration, and colony formation	[29]

### Digestive system

In gastric cancer, the expression of PCIF1 is significantly increased, which can be used as an independent predictor of poor prognosis. PCIF1 can modify TM9SF1 mRNA through m6Am and inhibit its mRNA translation, thus reducing the level of TM9SF1 protein, thereby promoting the development of gastric cancer [32]. The migration and invasion of the tumor can be prevented as well as the growth of the tumor and lung metastasis if PCIF1 expression is suppressed [32, 39]. According to studies, the circ-ATAD1/miR-140-3p/YY1/PCIF1 axis is essential for the growth of gastric cancer. The promoter domain of PCIF1 is attached directly by YY1, increasing transcription and advancing gastric cancer [40]. Xu found that the expression levels of PD-L1 and PD-1 in gastric cancer tissue were significantly increased and correlated with PCIF1 through bioinformatics analysis [41, 42].

In colorectal cancer(CRC), PCIF1 is upregulated and associated with poor prognosis. After inhibiting PCIF1, tumor proliferation, adhesion, migration and other malignant behaviors are improved [29]. PCIF1 controls the stability of FOS mRNA in colorectal cancer cells through m6Am deposition, thereby regulating TGF-β transcription. More interestingly, PCIF1 also has a certain effect on the tumor microenvironment. The number and specificity of natural killer(NK) cells in PCIF1 deficient tumors increased significantly, and the number and specificity of mononuclear MDSCs (M-MDSCs) decreased. Inhibition of PCIF1 improves the sensitivity of CRC tumors to PD-1 therapy by controlling PCIF1-mediated FOS-TGF- and Stat1/Ifitm3-IFN axis. The PCIF1-FOS-TGF-β pathway can be used not only as a target for cancer treatment, but also as a regulator of the ICB response. A LNPs packaging technique has been created by researchers to deliver chemically altered siRNA to tumors. The tumor growth of mice treated with LNP-PCIF1 siRNA was significantly restricted [29]. Some studies have shown that PCIF1 catalyzes the methylation of cap-Am in the nucleus, while FTO demethylates m6Am in the cytoplasm. Inhibition of PCIF1 can counteract the inhibitory effect of FTO overexpression on the CSC phenotype, thereby reducing the spheroidizing ability and chemosensitivity of cells [18].

In fatty liver induced by a high-fat diet, the PCIF1 index was significantly upregulated. Inhibition of PCIF1 can improve glucose tolerance, islet function and the development of nonalcoholic fatty liver [43].

### Urinary system

PCIF1 has an anti-tumor effect in bladder cancer. Studies on in vitro cells demonstrated that PCIF1 deletion significantly increased colony formation [44]. Additionally, bioinformatics research revealed a positive correlation between the expression of PCIF1 and the infiltration of CD4^+^ T cells in kidney clear cell carcinoma (KIRC) [42]. Additionally, it influences the majority of tumors' microsatellite instability and tumor mutation burden. However, there are no experimental data, and further verification is still needed [42, 44].

### Cardiovascular system

The main growth factor for numerous varieties of stem cells is called stem cell factor (SCF). According to prospective research, those with high SCF levels are less likely to experience fatal cardiovascular events and pass away[45]. The level of PCIF1 is significantly correlated with the level of plasma SCF, indicating that PCIF1 may play a significant role in cardiovascular events [46].

### Nervous system

The situation is different in glioma. Overexpression of PCIF1 inhibits cell proliferation, blocks the cell cycle of glioma at G2/M phase, and induces apoptosis. However, these effects may not completely depend on its methyltransferase activity but through binding to the WW domain [42, 47].

### Endocrine system

Claiborn proved that PCIF1 can target PDX-1 for ubiquitination and proteasome degradation [38]. Decreasing the dose of PCIF1 the gene can improve glucose tolerance and make the pancreas β normalize cell quality and save the Pdx1 ± mouse β apoptosis rate, normalizing the Pdx1 protein level and expression of the Pdx1 transcription target [48, 49].

### Motion system

There are many reasons for spinal canal stenosis, of which ligamentum flavum hypertrophy is the main cause [50]. He and others found that PCIF1 was differentially expressed in hypertrophy of ligamentum flavum, and further constructed a predictive diagnostic model for ligamentum flavum hypertrophy. PCIF1 may be a potential target for the infiltration of immune microenvironment with ligamentum flavum hypertrophy. However, it is still unclear how it regulates the immune infiltration [51].

### Infection

PCIF1 and m6Am modification also play an important role in various viruses. After HIV infection, the m6Am modification of cell mRNA decreased significantly, and the level of PCIF1 protein decreased significantly in a dose-dependent manner. The deletion of PCIF1 increases the expression of gp120 mRNA, thus inhibiting virus replication to limit HIV infection, and does not affect the packaging or release of HIV particles. PCIF1 can also inhibit HIV replication by enhancing the stability of the host m6Am gene (ETS1). Therefore, PCIF1 can be used as a replication inhibitor of HIV [31]. Some studies have shown that PCIF1 produces m6Am near the cap by modifying VSV mRNA. It can weaken interferon-β dependent antiviral responses. The m6Am methylation of viral mRNA protects the antiviral effect of the IFN-mediated innate immune response [52]. It has recently been shown that PCIF1 plays an important role in severe acute respiratory syndrome coronavirus 2 (SARS-2). PCIF1 enhances the mRNA stability of ACE2 and TMPRSS2 through m6Am modification to promote infection. This suggests that targeting m6Am may be a way to combat SARS-CoV-2 and other coronavirus infections.

## The role of PCIF1 in regulating tumor immunity

Tumor immunity has always been a hot topic of our attention, and the infiltration of immune cells in tumors is closely related to tumor prognosis. Currently, immunotherapy is being used more widely. Currently, there is little research on PCIF1 and immunity, but only a few articles have shown that PCIF1 plays an important role in tumor immunity and immunotherapy. Here, we summarize the current research on immunity with PCIF1/m6Am, which provides a new idea for future treatment directions.

Bioinformatics analysis has shown that there are differences in the expression of PCIF1 in various tumors [42]. In KIRC, PCIF1 is proportional to the infiltration of CD4^+^ T cells, neutrophils, MDSCs, and B cells. In thyroid cancer, PCIF1 is inversely connected with CD4^+^ T cells and positively correlated with the expression of macrophages and CD8^+^ T cells. There is a significant difference in the expression of PCIF1 in monocytes and macrophages in astroblastoma and sarcoma [42].

**Fig. 3:**
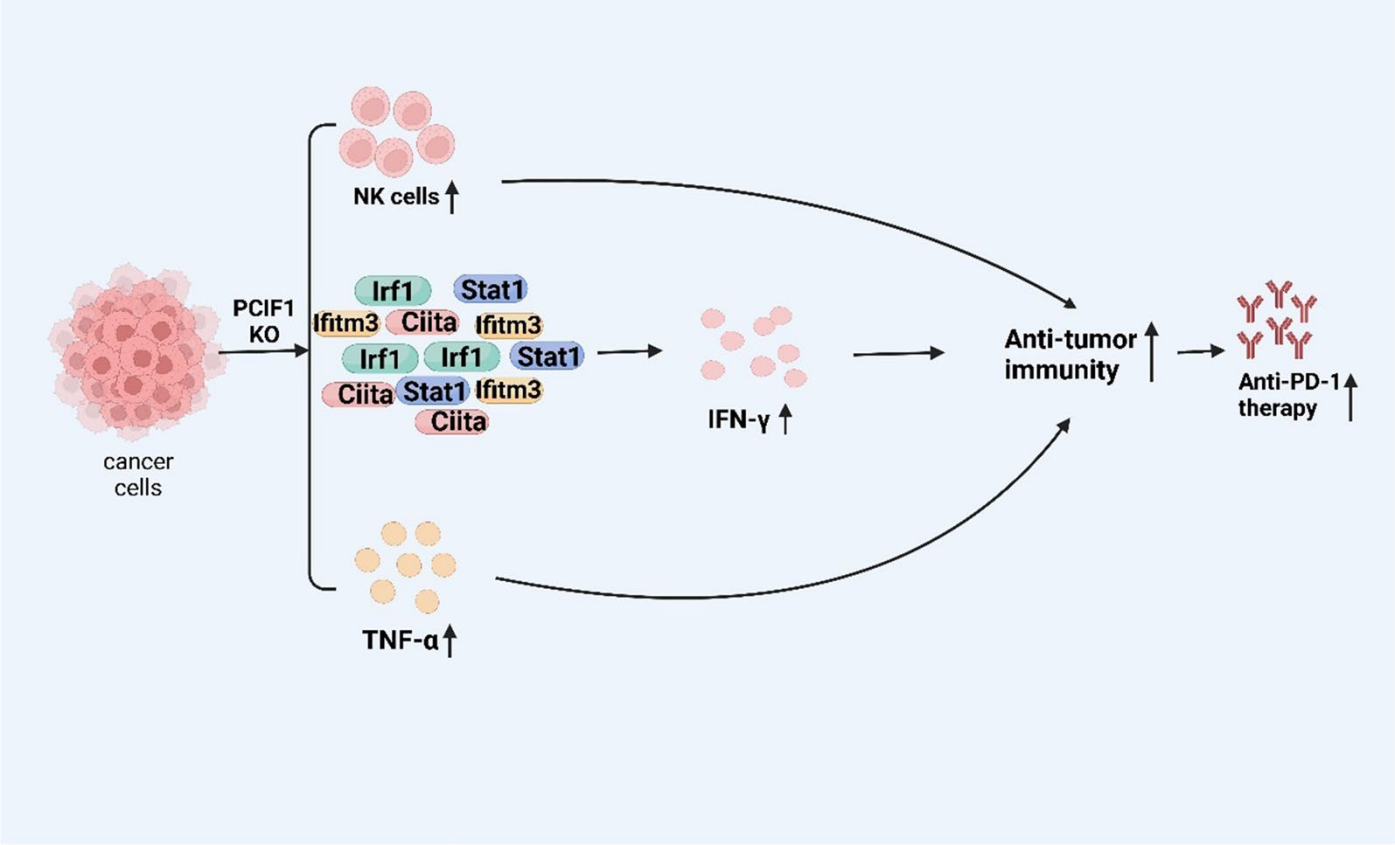
PCIF1 plays a crucial role in immunity and is associated with PD-1 treatment sensitivity

Researchers have also verified the relationship between PCIF1 and immunity in colorectal cancer [29]. Researchers used PCIF1 KO and control CT26 cells to form Subcutaneous tumor in nude mice and use flow cytometry to analyze the immune cell type in tumor tissue. The results showed that there were no differences in CD45^+^ lymphocytes, CD4^+^ T cells, CD8^+^ T cells, polymorphonuclear bone marrow derived inhibitory cells (PMN-MDSCs), or dendritic cells [29]. However, PCIF1 KO cells showed an increase in NK cells and a decrease in M-MDSCs. Such results were also observed using IHC staining. NK cells promote tumor immunity, while M-MDSCs inhibit tumor immunity. To distinguish which immune cells mediate the tumor suppression effect of PCIF1 KO cells, we conducted NK cell and mononuclear MDSC cell consumption on CT26 cells [29]. We observed that the antitumor effect of PCIF1 KO was completely reversed after NK cell consumption, indicating that NK cells were associated with PCIF1 KO mediated antitumor effects. RNA-seq was performed on PCIF1 KO cells and control cells, and it was found that genes were enriched in the innate immune response, cytokine response, and interferon-γ (IFN-γ) signaling. Irf1, Stat1, Ciita, and Ifitm3, which are involved in IFN-γ, showed significant increases after knocking down PCIF1, indicating that PCIF1 depletion enhances the IFN-γ reaction. In addition, TNF-α also increased after knocking out PCIF1. Both PCIF1 knockout and PD-1 therapy can inhibit tumor growth, but when combined, the tumor is almost completely eliminated. This indicates that PCIF1 knockout enhances the sensitivity of PD-1 and enhances its tumor killing ability [29]. These results suggest that PCIF1 is involved in the regulation of chemokines in the tumor microenvironment (Fig. 3).

## PCIF1 may be a tumor biomarker or target therapy in the future

Many studies have shown that PCIF1 plays an important role in tumors and other diseases, and PCIF1 is related to tumor immunity and immunotherapy. We speculate that PCIF1 can play an important role as a tumor biomarker and therapeutic target in the future.

Firstly, PCIF1 is generally differentially expressed in tumors. Bioinformatics analysis showed that there were differences in the expression of PCIF1 among 25 of 28 tumors [42]. In gastric cancer, PCIF1 protein will increase in the late stage. The level of PCIF1 in tumor tissue is higher than that adjacent to cancer, indicating that PCIF1 can not only reflect the presence of tumors, but also predict the stage in a dose-dependent manner [32]. Besides, PCIF1 is associated with prognosis. PCIF1 can be an independent prognostic factor in gastric and colorectal cancers [32]. Using Cox analysis, PCIF1 was identified as an independent adverse prognostic factor for gastric cancer. Studies have shown that among 27 patients with colorectal cancer, 19 patients with low PCIF1 expression have a significantly longer survival time than 8 patients with high PCIF1 expression, indicating that the dose of PCIF1 is also related prognosis [29]. Moreover, targeting PCIF1 leads to a decrease in malignant tumor behavior. In gastric cancer, colorectal cancer and bladder cancer, PCIF1 KO can reduce tumor migration, invasion and other malignant behaviors, reduce subcutaneous tumorigenesis, and even reduce lung metastasis, indicating that PCIF1 may be a target for tumor treatment in the future [29, 32, 44]. Researchers have developed a nanoparticles-mediated siRNA delivery system. siRNAs have been chemically modified to enhance their stability, lipophilicity, and cell permeability. This system can effectively knock out PCIF1. Then, siRNA was prepared in LNPs and injected into nude mice in the form of LNP-siRNA. The tumor size after treatment with LNP-PCIF1 siRNA was significantly reduced, indicating that inhibiting PCIF1 could improve tumor treatment. Finally, PCIF1 can be a target for immunotherapy [29]. PCIF1 KO can enhance sensitivity to PD-1 treatment in CRC tumors. PCIF1 KO has an impact on the tumor microenvironment, manifested by an increase in NK cells and a decrease in M-MDSCs, which also has an impact on immunotherapy [29].

However, there is still a long way from clinical application. Firstly, the role of PCIF1 in different tumors may be opposite. For example, PCIF1 is tumorigenic in gastric cancer, colorectal cancer, and bladder cancer, while it is a tumor suppressor in glioma [29, 47]. Secondly, it is difficult to determine the sample. The above studies are based on mRNA, protein, and tissue. We seek more easily accessible samples as biomarkers, such as blood and urine. There is currently a lack of such convenient research [32, 38].

## Discussion

m6Am modification is a ubiquitous modification in mRNA, that is catalyzed by the only methyltransferase in mammals. At present, there are only a few research-type articles indicating that PCIF1 has a clear correlation with tumor proliferation and development, and the physiological function of PCIF1 still needs further exploration. Here we summarize the structure of PCIF1 from the N-terminal to the C-terminal is a WW domain rich in tryptophan, a helical domain (HD), and an MTD, forming the "WW-linker-HD-MTD" structure. Moreover, we summarize scientific research progress of PCIF1 in recent years and find that PCIF1 plays an important role in transcription, maintaining mRNA stability and translation. More interestingly, PCIF1 may play a completely opposite role in different tumors. For example, PCIF1 is a poor prognostic factor in gastric cancer and colorectal cancer, while it plays an anti-tumor role in bladder cancer and glioma. The modification of PCIF1 on mRNA is also different. The researchers also observed that in MEL624 cells, PCIF1 inhibited post-transcriptional translation, but in HEK293T cells, it promoted translation. Bioinformatics analysis can also revealed that it plays an opposite role in different tumors. In addition, the existing research articles and the analysis of biographical data indicate that PCIF1 may be related to the therapeutic sensitivity of PD-1/PD-L1. In PCIF1 KO tumors, the immune cells in the tumor microenvironment changed, indicating that PCIF1 may be used as a therapeutic target for ICB in the future.

Since m6A and m6Am have no specific antibodies, it is difficult to distinguish between the common m6A-RIP. Researchers have developed a variety of specific measurement methods for m6Am, including m6Am-Exo-Seq, miCLIP, m6ACE-seq, m6Am-seq, CAPturAM and MeRIP-seq (m6A-seq). m6Am-Exo-Seq enables the elimination of uncapped mRNA fragments in the presence of nucleic acid exonucleases. The 5' end fragment of the capped RNA was then decapped to expose m6Am to enhance the recognition of m6Am by the m6A antibody, and the resulting RNA was immunoprecipitated and analyzed by sequencing [53, 54]. m6ACE-seq utilizes an anti-m6A antibody that photocrosslinks to m6A-containing RNA, thereby protecting m6A/m6Am as well as downstream RNA from 5’ to 3’ ribonucleases. This protected RNA was then sequenced to map RNA methylation at single base resolution. This method allows simultaneous mapping of m6A and m6Am [55]. CAPturAM is a method that does not require antibodies. The researchers utilized the cosubstrate miscibility of PCIF1 to install alkyne on its target, and used an affinity handle to enrich PCIF1 targets. The method has not yet been applied to transcripts [56]. m6Am-seq can directly distinguish between m6A and m6Am.The method relies heavily on in vitro methylation reactions. Enrichment of m6Am and removal of m6A by immunoprecipitation using cap-m7G resulted in over 100-fold enrichment of m6Am. Next, m6A IP was performed using m6A antibody. By comparing the methylation profiles of FTO-treated and non-FTO-treated samples, it was possible to specifically identify the m6Am site sensitive to methylase [53].

## Challenges, future directions and conclusion

There are still many questions to be explored in PCIF1.Firstly, the effect of PCIF1 on mRNA is still unclear. The regulation of transcription and translation is complex and multi-elemental. The specific mechanism of PCIF1-mediated m6Am modification on mRNA needs to be explored. Secondly, current methods of identifying m6Am still need to be advanced. Due to the lack of m6Am-specific antibodies, it is still possible to misidentify m6A as m6Am, resulting in false-positive results. Next, the relationship between PCIF1 and disease is currently unclear. Although several studies have shown its relevance to a variety of diseases such as gastric cancer, colorectal cancer, HIV and other diseases, many of them are cellular and animal experiments. There is a lack of authoritative clinical data to show the role of PCIF1 in diseases, which requires us to collect clinical data for further evidence. Finally, the relationship between PCIF1 and immunotherapy is unclear. Although immunotherapy is a hot topic of research nowadays, there are only few articles demonstrating the correlation between PCIF1 and PD-1.

In summary, although many physiological functions of m6Am are still unclear, it is undeniable that this is a universal and important post-transcriptional modification. The specific mechanism of PCIF1 in tumors in the future and how it affects the tumor microenvironment need further study.

## Data Availability

Not applicable.

## Abbreviations

m6A: N6-methyladenosine
m6Am: N6,2-O-dimethyladenosine
PCIF1: Phosphorylated CTD interaction factor 1
ICB: Immune checkpoint blockade
PD-1: Programmed cell death protein 1
m7G: 7-Methyl-guanosine
CTD: C-terminal domain
HD: Helical domain
pCTD: Phosphorylated C-terminal domain
HIF: Hypoxia-inducible factor
miRNA: MicroRNA
CRC: Colorectal cancer
NK: Natural killer
SCF: Stem cell factor
KIRC: Kidney clear cell carcinoma
